# On Medical Reform

**Published:** 1806-03

**Authors:** 


### *To the Editors of the Medical and Physical Journal.*

GENTLEMEN,

WHILE the business of medical reform only occupied the attention of the Lincolnshire Benevolent Medical Society, and the consideration of it was confined to a few obscure individuals in a remote part of a distant county, the subject appeared too ridiculous, and the promoters of it too inconsiderable to merit serious attention. But since this matter has been agitated in the metropolis; since plans have been handed about, sanctioned by the approbation of the College of Physicians; since it appears evident that such proceedings will lead to an increase of burthens on a profession already too much oppressed, it becomes an imperious duty on every member of that profession to consider well, before he sanctions by his approbation any deceitful measures of reform. I hope, therefore, to be excused for employing your pages in detecting the fallacy and iniquity of such proceeding; and if in the course of my observations, bold assertions should sometimes occur, I shall be careful that they are always such as may be supported by reference to facts, should necessity require it. However the medical profession has been stigmatized, and whatever may have been said of the degraded state of the practitioners of it, I shall assume as a fact, fact, that, taken collectively, no public body is to be found, which combines more learning, information, and industry; and that even among the lower professors of it, are to be selected men, whose natural abilities and acquired knowledge would not disgrace the list of the Royal College of Physicians. At the same time it must be allowed, that many persons have taken upon themselves the practice of medicine, without sufficient ability for so arduous an undertaking. This, however, has arisen from no fault in the profession, nor any neglect in the practitioners of it, but merely from the depraved state of the public opinion.

Medicine is an art dependent on public estimation; and those engaged in the practice of it, are more than any other set of men the creatures of fashion and caprice. If the voice of the multitude asserts that certain horse-doctors possess an universal knowledge of human diseases, as has been notoriously the case with the Taylors, the famous Whitworth doctors, what human power can prevent the credulous from submitting themselves, or their relations, to the care of such practitioners? And is it just that every medical man is to be taxed, because such is the folly of public opinion? If the powers of the strongest mind will not prevent such delusion, how can it be supposed that legal punishment would annihilate such pretenders to medical science?

Government is in the annual receipt of a considerable sum from the tax on patent medicines, which are sold in large quantities, with very little injury to the medical profession, and still less to the community, the nostrums being for the most part composed of ingredients too simple to occasion mischief. Is it then to be supposed that this lucrative branch of the revenue will be abandoned, or that the compounders of these nostrums, who frequently add M.D. to their names, are sufficiently under the law to be prevented promulgating their remedies? Many of the good people of England, and several even of those in exalted stations, would bear with equal impatience a government order for the abolition of theatrical amusements, as for the destruction of these worthy advocates for public esteem, who furnish the nervous invalid, and the debilitated rake, such abundant promises of returning health and renovated vigour. Perhaps it will be argued, that these are no reasons against a medical reform, and no refutation of the assertion, that the practice of medicine is in the hands of those who are incompetent: it is not contended tended that this assertion is yet answered, but the above stated facts are only adduced to show the difficulty, or indeed impossibility of restraining public opinion.

I next contend, that the very cause of this apparent degradation, is no other than an effort in the different members of the profession to maintain their respectability. It is notorious, that the few years of the last and present war have experienced an immense increase in every article of necessary consumption; and it is equally notorious that few, and those very inadequate advances, have been made by the medical profession, to enable them to meet the pressure of the times. People of reflection, viewing this circumstance, become unwilling to educate their children in a profession, whose toil is continual, and whose reward is poverty. The medical practitioner, obliged to have assistance in the compounding medicines, takes into his shop young men, whose toil repay their instruction while in his service, but whose previous education had been very inadequate to the study of the science of medicine; while medical students of better education, balancing in their minds the trifling difference between the expence incurred by a three years residence in Edinburgh, from whence they return with a degree of Doctor in Medicine, authorising them to take fees at each visit, and that of a two years residence in London, returning from whence they may visit a patient five times without receiving five farthings, are wisely led to turn their steps to the north, rather than to expose themselves to the humiliating condition of country apothecaries. Hence we find physicians in places where, formerly, the apothecary was the only medical adviser, except in desperate cases, when a physician was sought from a distance; and in places where formerly one physician resided, we have at this time two, three, or sometimes four. This consequence necessarily follows, the practice is insufficient to the support of the practitioners, and yet they must subsist; interested intimacies are formed with such members of the profession, as from ignorance or timidity are most likely to require their early assistance; the provinces of the surgeon and accoucheur are invaded, dispensaries are established, not merely for the purpose of supplying relief to the poor, but for the purpose of forming a connection and character, and occasionally furnishing medicine privately; the practice of sending prescriptions to druggists is encouraged; and druggists, or ill informed shopmen of apothecaries, are established in villages, under the the patronage of physicians, in order to promote the interest of the latter.

To remedy the evil complained of, I should therefore recommend a radical reform in the manners of the professsion; that they should mutually consider the situations of each other; that physicians, confining themselves to their particular province, should cease to interfere with surgery, &c. and only write where their prescriptions may be carried to the apothecary to be properly compounded, not trusted to the ignorance of a druggist, and the evil materials of his dirty shop; while apothecaries, by refusing to admit into their shops any other than youths properly prepared, would in time insure to the profession a respectable set of practitioners, equally unwilling to degrade the profession as to injure a brother practitioner.

I am, &c.

*Feb.* 14, 1806. VERITAS.*

P.S. I purpose, in a future communication, to offer some remarks on the Rules, said, in the Chirurgical Review, to be those circulated by the Royal College of Physicians.

* The Editors cannot admit future communications on this subject without the address of the Writer.

